# Defining competencies for the practice of telepsychiatry through an assessment of resident learning needs

**DOI:** 10.1186/s12909-016-0529-0

**Published:** 2016-01-26

**Authors:** Allison Crawford, Nadiya Sunderji, Jenna López, Sophie Soklaridis

**Affiliations:** Department of Psychiatry, University of Toronto, CAMH, Room 825, 250 College St., Toronto, Ontario M5T 1R8 Canada; Outreach and Telepsychiatry, Centre for Addiction and Mental Health, Toronto, Ontario Canada; Ambulatory Care, St Michael’s Hospital Mental Health and Addictions Service, Toronto, Ontario Canada; Department of Education, Centre for Addiction and Mental Health, Toronto, Ontario Canada

**Keywords:** Telepsychiatry, eHealth, Telemedicine, Psychiatry, Postgraduate medical education, Competency based medical education

## Abstract

**Background:**

A foundational assessment of learning needs is missing from previous reports of telepsychiatry curricula. We used an in-depth needs assessment to identify specific skills required for the practice of effective telepsychiatry, and provide an evidence base to guide the development of telepsychiatry curricula in postgraduate psychiatry training. Many of these skills set telepsychiatry apart from practice in traditional face-to-face clinical settings, or result from adaptations to clinical practice to meet the needs of a telepsychiatry interface in patient care.

**Methods:**

We used a qualitative, modified grounded theory approach to gain insight into areas of importance for telepsychiatry training in postgraduate psychiatry residency. 16 interviews of faculty and residents (9 and 7 interviews, respectively), allowed participants to reflect on their experiences in telepsychiatry. Data were then thematically analyzed.

**Results:**

Interview respondents identified important aspects of the context for telepsychiatry training; the skills required to competently practice telepsychiatry; and the desired teaching and learning methods for acquiring these skills. Specific domains of competency were identified: technical skills; assessment skills; relational skills and communication; collaborative and interprofessional skills; administrative skills; medico-legal skills; community psychiatry and community-specific knowledge; cultural psychiatry skills, including knowledge of Indigenous cultures; and, knowledge of health systems. The skills identified in this study map well to competency- based medical education frameworks.

**Conclusions:**

Telepsychiatry is increasingly being adopted as a solution to health systems problems such as regional disparities in access to care, and it requires explicit competency development. Ensuring adequate and quality exposure to telepsychiatry during residency training could positively impact our health systems and health equity.

## Background

Telepsychiatry, is the provision of mental healthcare at a distance through real-time videoconferencing, and may include direct assessment of a patient; **“**indirect care” of patients, such as through case consultation and supervision; education for healthcare learners, physicians and/or interprofessional healthcare providers; and program development. [1–6] Multiple studies demonstrate clinical outcomes for care via telepsychiatry that are equivalent to care delivered through traditional face-to-face methods. [7–10] Telepsychiatry is feasible to implement and sustain, and is rated as satisfactory, and sometimes preferable, to patients and family members across a variety of age ranges, clinical settings, and cultural populations. [6, 7, 11–13] Additional benefits include cost savings through limiting medical travel, better continuity and coordination of care, and early intervention. [6].

Advances in technology are progressively integrated into the practice of psychiatry, changing not only the nature of practice, but also the social reach of psychiatry, with increasing potential for timely access to care. Thus the impacts of telepsychiatry may be felt at every level of psychiatry from interpersonal and therapeutic communication, treatment modalities, to health systems delivery, and ethics. Because of the many benefits of telepsychiatry, particularly the potential to address health inequities that result from poor access to care, telepsychiatry use is likely to increase, and to take on evolving forms.

However, without formal exposure and education, psychiatrists may be hesitant to adopt telepsychiatry into their practice, particularly if they view telepsychiatry as an unfamiliar modality requiring specific technical and clinical skills. [14] Exposure during postgraduate training may lay a foundation that stimulates interest in telepsychiatry as a mode of practice, and provide the confidence and skills necessary to incorporate telepsychiatry into future practice. [5, 15]

Despite this need for exposure to telepsychiatry training during residency, there is limited literature to guide the development of sound pedagogical curricula. There are guidelines available to ensure best standards of care in telemedicine. The most commonly used guidelines in North America are those developed by the American Telemedicine Association, including guidelines specific to telemental health. [16] However, these guidelines do not provide guidance on how to impart these skills to trainees, or articulate underlying competencies. The need to identify educational standards and core competencies for training has been identified broadly in the field of telemedicine. Picot, for example, makes the case for, “the development and implementation of education and training standards, enabling professionals who practice [sic] in the field to obtain suitable skills, knowledge and recognition for telepractice.” [17] With competency-based education, we are beginning to see the emergence of e-Health competencies, including competencies specific to the practice of telemedicine [18, 19]. However, these have tended to focus on undergraduate education [19] and to be very general. Within the field of psychiatry such competencies have not been derived. In a recent literature review of telepsychiatry in graduate medical education, Sunderji, Crawford and Jovanovic identified only 20 peer-reviewed references that describe training psychiatry residents to provide mental health care through real-time videoconferencing. [14] Of these, only four delineate learning objectives, and none appear to have been derived from a rigorous assessment of residents’ learning needs. [14]

We believe that an assessment of resident learning needs is a necessary, and until now overlooked, foundation for developing evidence-informed approaches to training and curriculum development in telepsychiatry. Assessment of individual learner’s needs (individual needs and styles of learning), and of larger programmatic educational needs (curricular development; the interests or perceived needs of a whole target audience), is a crucial stage in planning the educational process. [20] It has been demonstrated that learning is more likely to lead to change in practice when needs assessment has been conducted. [20] Qualitative approaches to needs assessment can be most effective in attempting to understand how the area in question is understood by those concerned with it, or experiencing it, such as at initial or exploratory stages. [21] In telepsychiatry, this includes understanding how expert practitioners and learners at various stages of training understand the context and practice of telepsychiatry, the skills necessary to practice telepsychiatry, their own preparation and competency in necessary skills, and current and preferred learning methods. One validated method for getting at this information is to have clinicians and learners reflect on their own practical experience. [20] This can be aided by inviting “reflection of action,” or prompting respondents to draw on their experiential learning to identify innovations in practice, difficulties arising from practice, or gaps in knowledge or training. [20] Individual interviews have methodological rigor when trained interviewers are utilized, and allow more in-depth follow-up of responses and reflection on individual practice experience. [22, 23] For these reasons, we chose to use individual faculty and resident interviews to conduct the needs assessment.

We hypothesized that by reflecting on their previous learning and practice experiences in telepsychiatry, both residents and faculty would be able to identify specific skills required for the practice of effective telepsychiatry, including skills that set telepsychiatry apart from practice in traditional face-to-face clinical encounters. Expert users would be able to reflect more explicitly on aspects of their telepsychiatry practice than learning or novice users of telepsychiatry. Based on our prior literature review of telepsychiatry training in postgraduate medical education, we specifically wanted to understand learning needs across relevant domains, such as: i) technical; ii) collaborative/ interprofessional; and iii) administrative skills necessary for the practice of telepsychiatry. [14] We then planned to use these identified learning needs to articulate core competencies in the area of telepsychiatry and, if possible, to link these competencies to the array of pedagogical methods that may be deployed to build these competencies in learning practitioners.

## Methods

We undertook a qualitative study approach, drawing on methods from grounded theory, to explore residents’ learning needs with respect to telepsychiatry from both residents’ and faculty members’ perspectives. [24] In exploratory qualitative research, social phenomena are investigated with minimal a priori expectations in order to illuminate individuals’ experiences and develop expectations of those phenomena. [24] This study was reviewed and approved by the University of Toronto research ethics board.

### Expert reference panel

To ensure the relevance and usability of the findings of the study, we engaged a core team of 11 knowledge users representing Departmental administrators, leaders in education, telepsychiatry, education scholarship and residents. [25] The team met twice to shape data collection and interpretation, and apply the emerging findings toward: a) setting needs-based priorities (i.e., framed as competencies), and b) generating potential educational strategies geared toward resident acquisition of competencies. [26, 27]

### Recruitment

The study included University of Toronto Psychiatry resident participants who had expressed interest in telepsychiatry and/or education scholarship, as identified by the Expert Reference Panel.. Faculty member participants included those who are experienced in telepsychiatry as well as leaders in the University of Toronto, Department of Psychiatry’s strategic plan, divisions and teaching hospitals. Potential faculty member participants were identified both through the Expert Reference Panel. and through a previous environmental scan of telepsychiatry services in the department. We used purposive sampling to select information-rich cases that illuminated the research questions under study. Residents and faculty members were invited to participate in interviews through an email from either the first (AC) or second or NS author, followed up by an email from the interviewer (JL). Participation was voluntary and confidential, faculty and residents signed up by replying to the interviewer’s email.

### Data collection

Interviews were conducted by a research coordinator with expertise in qualitative research and interviewing. Academic researchers, such as research coordinators, without a health professional background can approach the interview without preconceived notions and can employ a certain degree of naivety to encourage thorough explanations from the interviewee. [28]

Interviews were conducted either by telephone or face-to-face depending on participant preference and availability. All interviews were audiorecorded and professionally transcribed. Participants followed an informed consent process, whereby consent to being interviewed and audio recorded was established verbally at the beginning of the interview and through a signed consent form.

In qualitative research, guidelines for determining non-probabilistic sample sizes are virtually non-existent. Thus, the researcher typically relies on the concept of *saturation*, that is, the point at which no new information or themes are observed in the data. For this study, we estimated we would need 10 resident and 10 faculty member participants. After 7 resident interviews and 9 faculty member interviews, we stopped seeking new participants as we had reached the point of saturation.

The interviews were semi-structured, with the interview guides serving as a framework of questions to be explored through discussion. Two interview guides were originally created: one for resident participants and one for faculty member participants. The guides focused on exploring the following themes: residents’ and faculty members’ perceptions of current and potential future telepsychiatry curricula, required competencies, barriers to gaining competencies, desired training opportunities, preferred learning methods, and attitudes toward technology in learning. While the interview guide for residents was implemented with success, a second iteration of the faculty member interview guide was needed partway through the study. After conducting a preliminary analysis of the first 5 faculty interview transcripts and consulting with the Expert Reference Panel., the research team found that the faculty participants were challenged when trying to access and articulate their knowledge and skills in telepsychiatry. It was difficult for them to describe several “taken for granted” tasks and explain their accumulated tacit knowledge of telepsychiatry. As a result, the research team, together with the Expert Reference Panel., revised the interview guide to include a cognitive walkthrough whereby the interviewee detailed the process of a typical telepsychiatry consultation during the interview. [29] For the remaining four faculty interviews, the research team found improvement in participant’s abilities to make explicit the required competencies for the practice of telepsychiatry.

### Data analysis

Interviews were audiorecorded and professionally transcribed verbatim. We discovered no differences between the telephone and face-to-face interview transcripts and therefore combined the results for analysis. The transcripts were analysed using the process of inductive coding. [30] All four authors independently read and made notes regarding potential themes, then, compared these notes for the first three transcripts. Through this comparison and discussion, a coding scheme was developed which was then applied to all the data with newly emerging codes discussed as needed. [31, 32]. The remaining transcripts were divided equally among three of the authors (AC, NS, and JL) and coded independently. Each theme or topic was organized into larger categories as the research proceeded. Finally, the codes were grouped together into themes with various subthemes which constituted the foundation of synthesising and conceptualising the data. A qualitative computer software package, NVivo, was used to store and organize the various codes derived from the data. [33]

## Results

### Sample characteristics

In total, 9 faculty members and 7 residents participated in interviews for this study. Most faculty member participants were male (*n* = 7, 78 %) while most resident members were female (*n* = 5, 71 %).

### Themes from qualitative interviews

Three broad thematic areas emerged from our data analysis: i.) *Context* for postgraduate training in telepsychiatry; ii.) *Competencies* to be achieved in telepsychiatry training; and, iii.) *Pedagogical approaches* to developing competence in telepsychiatry. More refined themes are grouped within each of these areas, below.

#### Context for postgraduate training in telepsychiatry

Respondents referenced many aspects of the context and parameters for the practice of and training in telepsychiatry. We grouped these into four broad clusters, including: health systems; perceived benefits of telepsychiatry; drivers for training in telepsychiatry, including rewards of practicing telepsychiatry; and, barriers to training in telepsychiatry.

All interviewees discussed the health systems context, including both the utility of offering care via telepsychiatry, and the expansion of and enhancement of the healthcare system through telepsychiatry. In particular interviewees viewed telepsychiatry as addressing gaps in the distribution of health human resources. One faculty participant stated,

*It’s an important tool in terms of dealing with the inequities in healthcare delivery in our province. So we have enormous resources in the big cities and the university-based towns. And very few resources outside of these so it’s a way of distributing our expertise in a more efficient way.* [Faculty – 208]

Several respondents highlighted the value to the health system of integrating care via telepsychiatry with care offered to communities through fly-in outreach. According to one faculty participant,

*The travel in Northern Ontario and work in the clinics there really helps with the telepsychiatry because often I’m doing a telehealth session for some community that I’ve visited or is not too different from the one’s I’ve visited. So I think that gives me a slightly richer understanding of the challenges and resources that the Northern communities have when I’m talking to clients.* [Faculty – 201].

Perceived benefits of telepsychiatry included equity, efficiency, economics and access. Faculty also listed the benefits to distant providers in providing support and reducing burn-out. Interviewees described drivers for training in telepsychiatry. The importance of champions was mentioned by several faculty respondents; for example,

*[For] any kind of program or initiative to really work, you know, we talked about obviously the time and the money and … probably the most important thing that has to be there and that’s the champions. And there have to be champions at you know, at a university and at a service delivery end, at a service recipient kind of end. If you don’t have the champions that are gonna take this on and push for this and advocate for it and make it happen, none of this stuff will happen.* [Faculty – 204]

Respondents also attributed personal connections to communities, either due to having lived or practiced there, as important personal drivers for doing telepsychiatry, and many cited social responsibility as both a motivation and a perceived benefit.

Faculty development was named as the biggest barrier to advancing training in telepsychiatry. One faculty participant stated, “A lot of faculty haven’t been involved in telepsychiatry video conferencing so again, there’s a lot of apprehension on their part. They definitely don’t feel prepared to do it themselves let alone supervise in that.” Several residents highlighted competing training demands. Another more implicit challenge that we identified was the tendency for some faculty practitioners to either assume that their practice via telepsychiatry is not differentiated from their approach to face-to-face care, or to have difficulty articulating the tacit knowledge and practice experience that they draw on. For example, a faculty participant stated,

*No, it’s exactly the same. The clinical work is the clinical work. You know, patients referred to you for a reason, you do your best to give a considered opinion and give some, do a consultation. Give a diagnosis. Give a treatment recommendation.* [Faculty – 206].

Another reflected on how s/he had just learned by ‘doing’ and questioned whether a special training program was required:

*I just did it. No, I think they can probably. This is a difficult question. It’s a different kind of work but I think if you’ve gone through the psychiatric training you adapt to the resources that are available or not available… I think it’s a get in there and work and do your work and figure it out kind of thing.* [Faculty – 202].

#### Competencies to be achieved in telepsychiatry training

Reflecting on their own practice, gaps in learning, and perceived learning needs, participants identified a wide-range of skills necessary for the practice of telepsychiatry. Many of these skills would also be more broadly applicable to the other areas of telemedicine practice. While some of these skills are also necessary in the practice of face-to-face psychiatry, each identified skill was understood as needing special consideration, practice, or adaptation within the telepsychiatry context. Guided in part by an earlier literature review [14], we grouped content into nine identified areas of skills utilized in the practice of telepsychiatry, and which participants articulated as being important to consider in designing training experiences, including: technical skills; assessment skills; relational skills and communication; collaborative and interprofessional skills; administrative skills; medico-legal skills; community psychiatry and community-specific knowledge; cultural psychiatry skills, including knowledge of Indigenous cultures; and, knowledge of health systems. These are summarized in Table 1, alongside related exemplary quotes from resident and faculty interviewees.

**Table 1:** Telepsychiatry competencies

Domain	Competency	Example quote
Medical Expert	Technical skills Operate equipment and software (cameras, recording, audio, etc.), optimizing use of their unique capabilities and trouble-shooting technical problems	*There’s kind of like a beginner level comfort level of telepsychiatry where you just kinda sit and talk to the TV but that’s all you do. And then there is actually setting up presets for all the people on the distant end and moving the camera and all that sort of stuff so you’re actually really making use of it, it’s not just a static looking at TV screen. Like I would follow kids around the room with the camera and I would, if I were doing case conference like 8 people, I set up 8 presets so I can zoom back and forth between different people… it takes full advantage of what you can do with the technology rather than it just being like Skype. [Faculty – 203]*
Medical Expert	Assessment Adapt the assessment process to be carried out at a distance: a. use distal partners to understand patient baseline and to conduct physical exams and lab work b. administer assessment tools such as the mini mental status exam c. conduct a comprehensive mental status exam via televideo (e.g., noting signs of substance intoxication and withdrawal) d. Integrate multiple streams and sources of information	*I was expecting it to be easier than it was… Even with the technology that’s being used, pretty high bandwidth pipe that’s available, there’s still this smidge of lag which is noticeable amazingly enough. And I think within psychiatry when you do an assessment, you’re paying particularly close attention to the response and timing and stuff like that. It’s fairly critical… I had to be kinda cognizant, wait an extra moment to see if it was a response or not and if the patient on the other end understood what I was asking or saying. And then the second thing kinda related is not being able to pick up on the body language and other cues as well as you would if the person is right in front of you in the room. [Resident – 102]*
Medical Expert	Medico-legal practices Adhere to legislation and professional regulatory standards (e.g., mental health certification, consent & capacity law, and privacy law) while providing care at a distance	*especially this thing about medical, legal, and risk issues, that’s something that hasn’t come up in my experience with telepsychiatry but I’d imagine that would be a bit anxiety-provoking for both sides, you know when someone is suicidal or there is, you know, a safety issue, how do you address that when you’re not really in the room? So I would think that would require teaching and maybe extra skills, how to navigate that. [Resident – 106]*
Communicator	Conduct a psychiatric interview over technology, including forming a therapeutic alliance, demonstrating flexibility in the structure of interview, completing the full interview in the allotted time, and where appropriate incorporating family members	*I think it’s overall you have to be more active in telepsychiatry. It’s not like a kind of passive role. You have to really put a little more effort into it I think than doing it face to face. [Faculty – 206]*
	Craft a useful consultation note, relevant to the community, context and resources	*So I think clinically or interpersonally, it requires an ability to form a really rapid therapeutic alliance with the person. Because you’re only gonna see them that one time and you only have a limited amount of time to get through what you need to do to do a proper assessment so I would say that, you know, you really, if you don’t have those types of skills to seem competent in what you’re doing and be approachable and open to questions whatever, that would make it pretty difficult. [Faculty – 207]*
Collaborator	Work with interprofessional providers across distance, using them to better understand the community and available resources, and to achieve continuity of care over time Engage in collaborative models and “indirect care” through telepsychiatry to reach larger numbers of patient and families Coordinate care beyond the healthcare system (e.g., education system, social assistance system, child protection, etc.)	*Consultations are fine for some things but not for everything. And where the consultative model falls apart is if in an ongoing kind of relationship, patients change over time. They’re not the same just because you saw them once. So collaborative care allows that ongoing relationship and continuity of care working through the primary clinician to keep supporting them as they keep doing that… the bottom line is telepsychiatry or any service from a distance by definition has to be a collaborative care service because we’re not there on the ground providing the primary care. [Faculty – 204]*
Manager	Administrative abilities Understand how the technology network is organized, accessed and administered at the proximal and distal ends	*There’s a process. It’s coordinated by the hospital or hospitals have a local OTN coordination service and my administrative coordinator had to learn how to book them into through the hospital appointment booking program because the patients do get rostered into our electronic medical record with a chart. And a code that shows that the service was provided by telemedicine. So it’s sort of an added layer of complexity…[has] a reasonable amount of impact on a department’s ability to provide this service in high volumes…Not insignificant increase in the administrative burden of coordination. [Faculty – 209]*
Advocate	Community and cultural psychiatry Appreciate the social determinants of health in distal communities that may influence patient presentation (e.g., large scale layoffs; community event or tragedy)	*I wouldn’t presume to know like something about any given community simply because I’ve been somewhere else nearby geographically…What’s actually the structure of the service I’m working with so, is it a family health team? Who’s actually on site? What kinds of things are actually available to patients there? …Because you don’t want to make a recommendation that’s far out strips what can actually be provided. And I want to sort of be aware of sort of what’s there, their knowledge of their community. They may or may not know about some other things that are available in the community. [Faculty – 209]*
	Demonstrate awareness of/sensitivity to local cultures, beliefs, knowledge systems, resources, healing practices, and views of technology	*cultural and social consideration in telepsychiatry. I think it’s a really important topic;…How sitting in front of a video screen would be different for someone in the Inuit First Nations population…[Resident – 102]*
	Reflect on one’s own social position coming from an academic, typically urban or regional centre	*I see a lot of First Nations folks through telepsychiatry… different aspects of their care…whether exploring whether or not they are interested in not just western-based medical practices but traditional-based, you know, healing activities like ceremonies, traditional medicines…not really knowing if someone has access to that. But also not assuming just because someone as an example is Aboriginal is that they’re necessarily going to want to see a traditional healer. [Faculty – 207]*
Advocate	Health systems Use technology to address health human resource problems and systemic inequities in access to care Promote and respond to the health needs of individual patients, families and communities.	*And then better efficiency. We have an incredibly inefficient sort of ambulatory psychiatric system and this sort of has the potential to change that a bit. I don’t think it can take over all of psych care. I do think there’s something to be said about in-person care for some specific cases but I do think it can certainly redistribute sort of vast inequity in the accessibility that’s sort of a geographic barrier more than anything else. [Faculty – 209]*
		*I think there’s a huge potential with telepsychiatry and collaborative care to be an advocate… to talk to the stakeholders… So I’d even emphasize the potential for health advocacy. [Resident – 106]*

#### Pedagogical approaches to becoming competent in telepsychiatry

Participants made a number of recommendations for consideration when designing curricula in telepsychiatry, which we grouped according to: experiential learning; didactic learning or group learning; embedded in rotation; mandatory versus elective educational opportunities; timing; and “dose” or quantity of exposure. All participants listed experiential or ‘hands on’ learning as critical for acquiring the necessary skills in telepsychiatry. As one faculty participant emphatically summarized:

*Hands-on. Definitely hands-on. Workshops limited… As much as people can get experience or exposure… and get that experience for themselves firsthand…You know, kind of a picture is worth a thousand words but I don’t know, a video connection would be worth 10, 000 or something… People can learn much more about it by just coming and observing and participating than I can do it justice by just talking about it… unless someone is actually there and actively involved and seeing it, participating, they may not actually see the relevance for themselves and won’t maintain the interest in it.* [Faculty 204].

The importance of faculty expertise was again noted, in relation to experiential learning:

*I think the experiential component is the most important part. Ideally with a supervisor who’s got a significant comfort level with the technology. So that like with any clinical modality, you want someone to help supervise the resident who themselves has a degree of expertise in the area. If they’re fumbling with it or if they’re frustrated with it then that’s not gonna do much for the resident’s own comfort level.* [Faculty 203]

However, most participants also expressed that there is a role for didactic learning in a group format, particularly for information such as the evidence-based literature supporting telepsychiatry. Several resident participants expressed an interest in small group formats (of 3–4 learners), where the didactic material could be integrated with experiential learning through discussion. There was unanimous agreement that telepsychiatry should be introduced early in psychiatry training, with opportunities at all stages of training, across different clinical populations and contexts (eg., general psychiatry, child psychiatry, geriatric psychiatry, consult liaison psychiatry, hospital-based and community settings, etc.), and allowing for graduated responsibility. Competency was understood as being achieved through iterative practice. All participants agreed that some exposure to telepsychiatry should be provided and expected, but were divided on whether this should be a mandatory part of training.

## Discussion

This approach to identifying resident learning needs in the area of telepsychiatry has several limitations. We only drew on resident and faculty respondents from one institution, although our program is the largest postgraduate psychiatry training program in North America, and does a sizeable volume of outreach to remote and underserviced areas, both in person and via telepsychiatry. Second, this needs assessment captures “felt needs” (i.e., what people say they need), and normative needs (i.e., identified by experts), in contrast to other kinds of learning needs. [20] Related to this, expert practitioners interviewed for this study experienced some difficulty articulating specific skills that are often tacit. While all faculty participants were supportive of introducing some educational opportunity in telepsychiatry for residents, many had difficulty making explicit their skills and the underlying competencies necessary for the effective practice of telepsychiatry. In other words, it was difficult for them to identify what made them an expert, and yet both faculty and residents alike noted the importance of experiential learning with supervision by experts. Additional methods of needs assessment, such as ethnography or simulation could achieve a more complete picture of the skills involved in providing telepsychiatry. Any pedagogical approach for trainees at all levels should also incorporate existing practice guidelines available at International [34], National [16] and regional levels, as well as medicolegal parameters established by the practitioners’ governing body. In turn, the ongoing development of telemedicine guidelines should incorporate discussion of underlying competencies required to deliver the best care [35]. Finally, learning and development of competency is only one barrier to the uptake of telepsychiatry; other barriers, such as administrative support, societal values, recruitment of faculty, and models of remuneration, were beyond the scope of this study.

Notwithstanding these limitations, both resident and faculty interview respondents were able to describe a rich array of skills, across many domains of competency, that they either relied on, or wanted exposure to through educational curricula in telepsychiatry. This study is the first primary research that we are aware of to rigorously explore learning needs in telepsychiatry. Our use of qualitative methods, with a focus on reflection on action or practice, ensured that we did not constrain the kinds of responses that interviewees provided, instead allowing for a wide-ranging exploration across domains of practice. In relation to the limited literature that described telepsychiatry curricula, our results aligned most closely with the curriculum advanced by Oesterheld and colleagues, to the extent that we identified skills that extend beyond technical skills, including assessment skills, and adaptation of skills to the telepsychiatry context. [36] However, through an earlier literature review [14] we considered areas of practice that Oesterheld’s earlier curriculum did not explicitly address, including collaborative and interprofessional skills, community psychiatry, cultural psychiatry, medico-legal aspects, and health systems. The current study allowed us to more specifically identify these areas of telepyschiatry practice that should be addressed in a telepsychiatry curriculum.

Delineating skills and competencies across domains of practice also aligns with current frameworks and priorities in postgraduate medical education, which are increasingly oriented toward demonstrable skills and the intended outcomes of training. [37] Competency- or outcome based medical education could provide an advantageous framework for curriculum design in telepsychiatry, to ensure clarity and relevance of competencies, and to encourage accountability of training institutions to train residents. [38–40] Many of the skills identified by participants in this study readily map onto objectives within existing competency-based frameworks in postgraduate medical education. Competencies in patient care, medical knowledge, interpersonal communication, professionalism, and systems-based practice, for example, are referenced by the Accreditation Council for Graduate Medical Education in the USA. [41] Within our own Canadian learning context, the telepsychiatry skills identified through this study map onto the CanMEDS roles, particularly the roles of medical expert, communicator, collaborator, manager (changing in the 2015 framework to Leader), and health advocate. [42] CanMEDS has been adopted by countries on five continents, including the Royal College of Physician and Surgeons of Canada, Royal College of Psychiatrists in the UK, and Royal Australian and New Zealand College of Physicians, making it the world’s most recognized and most widely applied physician competency framework [37]. The first column of Table 1 aligns the skills identified by our participants with the CanMEDS framework.

Many of the skills identified by participants may thus be understood as applicable across psychiatry, rather than as competencies unique to telepsychiatry. We argue that although these skills may be subsumed under general categories of competence (such as communication), we have identified unique applications of these competency areas specific to telepsychiatry. This is similar to the approach taken by the eHealth Expert Working Group advising the development of the CanMEDS 2015 framework. Instead of creating a new or “role” devoted to eHealth, the working group looked at each of the CanMEDS Roles through the lens of eHealth. [18] However, they broadly considered eHealth, including electronic health records, and information technologies, and broad ethical issues such as ‘big data’ and surveillance, and do not consider telepsychiatry and telemedicine specifically.

Finally, our study also begins to illuminate the pedagogical methods preferred by teachers and learners for the development of competency in the practice of telepsychiatry. We can continue to draw on effective programs in telepsychiatry education, such as the national program successfully developed in the US [43], while making explicit the competencies addressed in such programs, and matching the best methods to specific competencies. In our interviews, experiential learning is understood as critical, with graduated exposure and increasing responsibility, starting early in training, supported by the majority of respondents. In our own institution we have used the results of this needs assessment to create a telepsychiatry curriculum that spans different stages of training, supporting the acquisition of the above-named competencies through a blend of didactic, case-based and experiential learning of telepsychiatry across the lifespan, general psychiatry, and subspecialty areas. Faculty are supported to mentor learners in the acquisition of specific skills through a faculty tip sheet, and goals and objectives and summative evaluations that target these specific competency areas.

## Conclusion

We hypothesized that by reflecting on their previous learning and practice experiences in telepsychiatry, both residents and faculty would be able to identify specific skills required for the practice of effective telepsychiatry, including skills that set telepsychiatry apart from practice in traditional face-to-face clinical encounters. The needs assessment supported this, and identified telepsychiatry specific skills and/or applications in the areas of technical skills; assessment skills; relational skills and communication; collaborative and interprofessional skills; administrative skills; medico-legal skills; community psychiatry and community-specific knowledge; cultural psychiatry skills, including knowledge of Indigenous cultures; and, knowledge of health systems. We mapped these onto the competency-based CanMEDS framework. This needs assessment provides an evidence-base to inform development of curricula, including content and pedagogical methods, which can then be evaluated based on the degree to which it allows residents to achieve the necessary competencies, In addition to validating the need for specific training in telepsychiatry, this needs assessment also emphasizes the importance of faculty development in order to champion telepsychiatry practice, and to provide faculty with the ability to articulate the skills embedded in their practice. If we can translate this knowledge to residents, we stand to favorably influence their future practice and the reach of psychiatry. As one resident participant stated,

*Yeah, I don’t think I’d want to work in a very rural, remote place, but I’d be very interested in doing what I’ve seen where staff might live in [name of city], and then have kind of like a sister practice in a sense where they work and help and coordinate with them. [Resident 101]*
